# Modulating the tumor microenvironment via oncolytic virus and PI3K inhibition synergistically restores immune checkpoint therapy response in PTEN-deficient glioblastoma

**DOI:** 10.1038/s41392-021-00609-0

**Published:** 2021-07-28

**Authors:** Fan Xing, Jingshu Xiao, Junyu Wu, Jiaming Liang, Xiaoyu Lu, Liping Guo, Ping Li, Panpan Hou, Chunmei Li, Deyin Guo

**Affiliations:** MOE Key Laboratory of Tropical Disease Control, Centre for Infection and Immunity Study (CIIS), School of Medicine, Sun Yat-sen University, Shenzhen, China

**Keywords:** Immunotherapy, Cancer therapy, CNS cancer

**Dear Editor**,

Glioblastoma (GBM) is a lethal primary brain cancer, with a median survival of less than 2 years.^1^ Immune checkpoint blockades (ICBs) have revolutionized cancer therapy in the last decade, but they have little clinical benefit in GBM.^1^ Genomic and transcriptomic analysis revealed a significant enrichment of PTEN mutations in GBM patients resistant to ICBs.^2^ PTEN deficiency activates the phosphatidylinositol 3-kinase (PI3K)-AKT pathway to shape an immunosuppressive microenvironment.^3^ However combination of PI3K inhibitor and PD-1 blockade only has moderate synergetic effect in PTEN-deficient tumor, as the combination does not induce tumor regression.^3^ Therefore, overcoming the resistance to ICBs in PTEN-deficient GBM remains a clinical challenge.

Recently, oncolytic virus (OV) has been recognized as an attractive cancer treatment.^4^ Our group has previously made a breakthrough in the antiviral function of PTEN,^5^ in which the disruption of PTEN function improves viral infection.^5^ Thus, we hypothesize that the PTEN-deficient GBM may turn to be a promising target for OV therapy. In this study, we try to use OV treatment to improve the efficacy of the former combination of ICB and PI3K-AKT pathway inhibition.

In order to explore a possible therapeutic route to overcome the resistance to ICB treatment in PTEN-deficient GBM, we established a PTEN-knockdown GL261 cell line using small hairpin RNA (shRNA) and engineered an orthotopically xenografted model in C57 mice. As expected, anti-PD-1 treatment inhibited tumor growth and prolonged the survival time of mice inoculated with GL261-shNC, but not in mice with GL261-shPTEN (Fig. 1a and Supplementary Fig. 1a). PTEN deficiency also decreased the number of CD8^+^ T cells in the GL261-shPTEN mice (Supplementary Fig. 1b), which is similar to clinical observations of GBM patients with PTEN mutation.

**Fig. 1:**
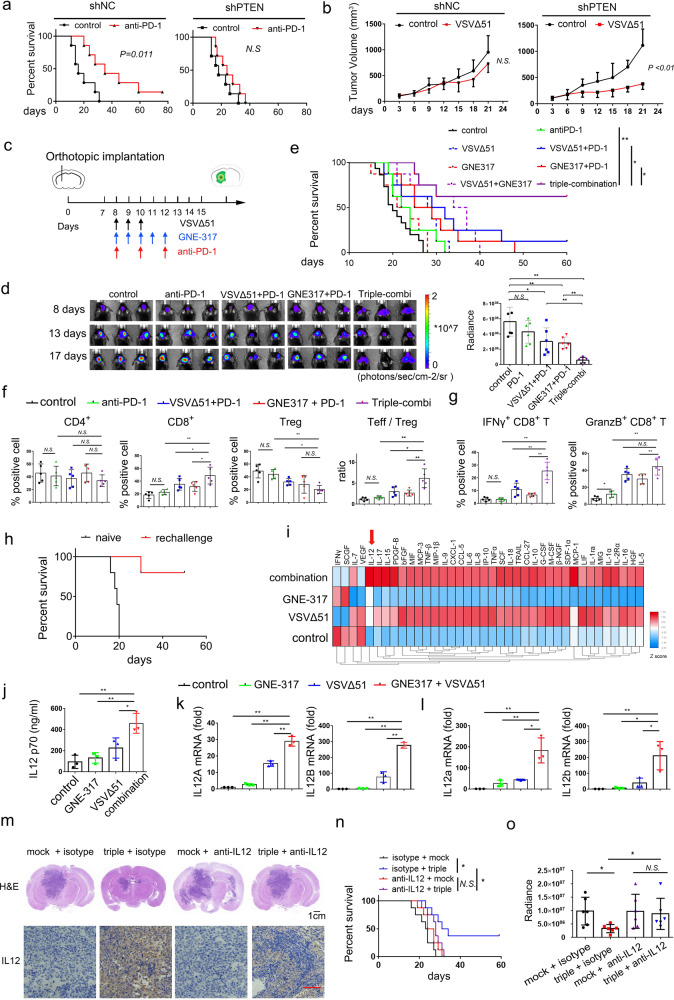
**a** Knockdown of PTEN led to resistance to anti-PD-1 therapy. C57 mice were implanted with 3*10^5^ GL261-shNC or GL261-shPTEN cells on day 0. Then, the mice were treated with anti-PD-1 antibody (10 mg/kg) at day 8th, 10th, and 12th. The survival times of mouse was monitored. **b** Knockdown of PTEN increased the sensitivity to OV. Nude mice were implanted with 3 × 10^6^ GSC1-shNC or GSC1-shPTEN cells on day 0. Then, the mice were treated with 3 × 10^7^ PFU VSV on day 5 through 7. **c** The timeline of drugs delivery. **d** Left panel: tumor growth was monitored via bioluminescence imaging of luciferase activity in GL261-shPTEN xenograft mice (*n* = 6 per group) on day 8th, 13th, and 17th. Right panel: quantitative radiance of mice on day 17 was analyzed. **e** Kaplan–Meier survival curve of mice bearing GL261-shPTEN1 tumors. **f** Percentages of CD4^+^ T cells, CD8^+^ T cells among CD45^+^ cells and Tregs (Foxp3^+^) among CD4^+^ T cells were analyzed in brain tumor tissue. **g** The function of CD8^+^ T cells was evaluated by measuring IFNγ and granzyme B. **h** The survived mice (*n* = 5) from the triple combination experiment were rechallenged on day 80 with a 2-fold increase in the number of GL261-shPTEN cells (6 × 10^5^). Naïve mice of similar age (3 months) were implanted as controls (*n* = 5). **i** Cytokine array screening of the conditioned medium of U251 cells by Luminex technology (Plex pro Human Cytokine). Cells were treated with GNE317 (1 μM), VSVΔ51 (MOI = 0.1) or the combination for 24 h. **j** ELISA analysis of IL-12 release in U251 cells. **k**, **l** qRT-PCR analysis of mRNA levels of IL12A and IL12B in U251 cells (**k**) and in GL261 cells (**l**). **m** Depletion antibody against IL-12 was injected to reduce the glioma derived IL-12. The mice were euthanized and subjected to H&E staining (scale bar = 1 cm) to detect tumor growth and immunohistochemistry (scale bar = 100 μm) to evaluate the expression of IL-12 on fifteen days after tumor inoculation. **n** Kaplan–Meier survival curve of mice. **o** Monitoring of tumor growth via bioluminescence imaging of luciferase activity in mice (*n* = 6 per group). Quantitative radiance of mice on day 14 was analyzed. All quantitative data are presented as mean±SD. [**p < 0.01; *p < 0.05; *N.S*. no significant differences, log-rank test (**a**, **b**, **d**, **h**, and **n**), one-way ANOVA (**e**–**g**, **j**–**l**)]

We next probed the effect of PTEN deficiency on the response to OV treatment. In the subcutaneous xenografts of nude mice, the treatment of VSVΔ51, an engineered OV strain from vesicular stomatitis virus (VSV), significantly inhibited tumor growth and induced apoptosis in PTEN-deficient xenografts, but not in PTEN-intact ones (Fig. 1b and Supplementary Fig. 1c). Knockdown of PTEN enhanced the tumor-selective replication and tropism of OV in a bilateral tumor model (Supplementary Fig. 1d). We further confirmed that PTEN could regulate replication and oncolysis of OV in different glioma cell lines (Supplementary Fig. 1e–m). Mechanistically, PTEN regulated the type I interferon pathway via PI3K-independent way (Supplementary Fig. 2a–f). We found that PI3K inhibitor did not influence the infection and proliferation of OV in PTEN-deficient cells (Supplementary Fig. 2g, h). Collectively, the differential responses to PD-1 blockade and OV treatment in PTEN-deficient GBM drives us to explore the effect of the combination therapy.

In light of our findings, we investigated whether the addition of OV could improve the moderate effect of the PD-1 blockade and PI3K inhibitor in PTEN-deficient tumor. We used GNE-317 as PI3K-specific inhibitor, as it is a blood-brain-barrier penetrative drug. The mice orthotopically transplanted with GL261-shPTEN tumors were treated with (i) anti-PD-1 antibody; (ii) GNE-317; (iii) GNE-317 and anti-PD-1; (iv) VSV∆51; (v) VSV∆51 and anti-PD-1; (vi) GNE-317 and VSV∆51; (vii) triple-combination with GNE-317, VSV∆51, and anti-PD-1 (Fig. 1c). The growth of GBM xenografts was monitored using bioluminescence in vivo imaging systems over the course of the experiment (Fig. 1d). Dual combination of VSV∆51 or GNE-317 with anti-PD-1 slightly enhanced the survival time of mice, as there is few long-term survived mice (Fig. 1e). Strikingly, triple-combination treatment induced tumor regression and improved survival with complete tumor eradication in five of eight (62%) mice (Fig. 1e).

To systematically profile the immune cells infiltrated into tumor, we conducted flow cytometric analysis (Supplementary Fig. 3a). The triple-combination treatment increased the percentage of CD8^+^ effector T cells (Teffs) and lowered the quantity of regulatory T cells (Tregs), leading to the increased ratio of Teffs to Tregs (Fig. 1f and Supplementary Fig. 3b). Triple combination therapy also significantly increased both interferon-γ and granzyme-B positive CD8^+^ T cells, compared to other treatments (Fig. 1g and Supplementary Fig. 3c). We further investigated that immune cells mediated the antitumor activity through in vivo depletion experiments by using antibodies against CD4^+^ and CD8^+^ T cells and liposomal clodronate against macrophages. Depletion studies demonstrated that tumor regression was dependent on CD8^+^ T cells and CD4^+^ T cells, but not macrophages (Supplementary Fig. 4a–f).

There is no observable change in body weight (Supplementary Fig. 5a). Histological analyses of vital tissues displayed no significant abnormal pathological change (Supplementary Fig. 5b). Long-term survivors were re-challenged on day 80 with a 2-fold increased glioma cells. Most of re-challenged mice did not succumb to the new tumors, while all age-matched naïve mice succumbed to tumors by day 20 (Fig. 1h). These results indicated that combination of OV and PI3K inhibitor synergistically and safely restored the efficacy of anti-PD-1 treatment, and established long-term antitumor immunity in PTEN-deficient GBM model.

We next explored the synergistic mechanism of the combination therapy. We found that the conditioned media from U251 cells (PTEN-deficient) treated with GNE-317 and VSV∆51 combination could induce IFN-γ expression in Jurkat-T cells (Supplementary Fig. 6a, b), suggesting the release of chemokines or cytokines from glioma cells upon the treatment. Cytokine array analysis revealed that IL-12 was markedly induced upon combination treatment compared to VSV∆51 or GNE-317 single treatment (Fig. 1i). Consistently, the release of IL-12 and mRNA expression of *IL12A/12B* significantly increased following the combination treatment (Fig. 1j–l and Supplementary Fig. 6c). The combination also synergistically increased the expression of IL-12 in brain tumor (Supplementary Fig. 6d) and the ratio of Teff cells to Treg cells (Supplementary Fig. 6e, f).

Furthermore, either knockdown of *IL12* in glioma cells or blocking of IL-12 with a neutralizing antibody eliminated the activation of Jurkat-T cells by the glioma cell conditioned medium (Supplementary Fig. 7a–d). Taken together, PI3K inhibitor cooperates with OV to reshape the tumor microenvironment by inducing glioma-derived IL-12 production and stimulating T cells.

To evaluate the role of glioma-derived IL-12 in modulating the antitumor effect of the triple combination therapy in vivo, we eliminated glioma-derived IL-12 induction by using neutralizing antibody or knocking down expression of *IL12* by shRNA. We found that either antibody depletion or knockdown of *IL12* decreased the IL-12 upregulation, and the anti-tumor effect elicited by the triple-combination in vivo (Fig. 1m and Supplementary Fig. 8a, b). Kaplan–Meier analysis revealed that antibody depletion and knockdown of *IL12* could abolish survival benefit gained from the triple-combination (Fig. 1n and Supplementary Fig. 8c, d). This is corroborated by in vivo bioluminescence-based image analysis (Fig. 1o and Supplementary Fig. 8e, f). These data suggest that IL-12 derived from glioma cells mediated the antitumor effect and tumor regression in the triple-combination therapy.

In summary, this study introduced a rational design of “chemical/biological/immunological” triple combination therapy to overcome the immuno-resistance of PTEN-deficient GBM (Supplementary Fig. 9). The triple-combination of OV, PI3K inhibitor, and anti-PD-1 boosted antitumor immunity, eradicated tumors in the most of mice and established long-term antitumor immune memory. Mechanistically, OV treatment cooperated with PI3K inhibitor to remodel the glioma-derived cytokine pattern by enhancing the secretion of IL-12, which subsequently activated T cell function and reshaped the immunosuppressive state. Our results identify PTEN deficiency as the biomarker of OV therapy and indicate that targeting tumor-derived cytokine profiles is an attractive strategy to modulate the tumor microenvironment. As PTEN deficiency is around 40% in GBM, our study has clear implications for GBM therapy.

## Supplementary information

Supplementary
