# Supplementary material for: Modulating the tumor microenvironment via oncolytic virus and PI3K inhibition synergistically restores immune checkpoint therapy response in PTEN-deficient glioblastoma
Source: Signal Transduct Target Ther. 2021 Jul 28;6:275. doi: 10.1038/s41392-021-00609-0 (PMC8316409; doi:10.1038/s41392-021-00609-0)
Supplement: Supplementary file 1 — Supplementary [file 41392_2021_609_MOESM1_ESM.docx]

Supplementary Materials for

**Oncolytic Virus and PI3K Inhibition Synergistically Restores Immunotherapy Response in PTEN-Deficient Glioblastoma**

**Author information**

Fan Xing^1,2^, Jingshu Xiao^1,2^ , Junyu Wu^1,2^, Jiaming Liang^1^, Xiaoyu Lu^1^, Liping Guo^1^, Ping Li^1^, Panpan Hou^1^, Chunmei Li^1^&Deyin Guo^1,*^

^1^MOE Key Laboratory of Tropical Disease Control, Centre for Infection and Immunity Study (CIIS), School of Medicine, Sun Yat-sen University, Shenzhen 518107, China.

^2^ These authors contributed equally to this work.

*Correspondence to: [guodeyin@mail.sysu.edu.cn](mailto:guodeyin@mail.sysu.edu.cn)

Supplementary Materials:

Materials and Methods

Supplementary information Fig. S1-S9

Supplementary information, Table S1 and S2

**Materials and Methods**

**Cells**

U251, Jurkat cell lines were purchased from the American Type Culture Collection. They are maintained at 37°C under 5% CO^2^ in high glucose DMEM supplemented with 10% [fetal bovine serum](https://www.sciencedirect.com/topics/neuroscience/fetal-bovine-serum) (Gibco) and penicillin/streptomycin. The mouse glioma cell line GL261 and primary patient-derived GSC1 were provided by Dr. Guangmei Yan (Sun Yat-sen University). GL261 cells are cultured in DMEM/F12 medium supplemented with 10% [fetal bovine serum](https://www.sciencedirect.com/topics/neuroscience/fetal-bovine-serum) (Gibco). GSC1 is cultured as previously described.[^1^](#_ENREF_1) Briefly, GSC1 cells are cultured in DMEM/F12 medium supplemented with 2% B27 (Gibco), 0.5% penicillin G-streptomycin sulfate-amphotericin B complex (Corning), recombinant human EGF (20 ng/mL; R&D Systems), and recombinant human FGF-basic (20 ng/ml; Peprotech). Spheres were dissociated with Accutase (Innovative Cell Technologies) for passaging.

**Lentivirus-mediated gene knockdown, gene transfer and stable cell lines construction**

Mission pLKO.1 lentivirus vector with puromycin resistance gene containing shRNA-luciferase (shPTEN1-luc，shPTEN2-luc), pLKO.1-puro lentivirus vector with blasticidin resistance gene containing shRNA (shIL12A), the nonmammalian shRNA control, phage-PTEN, phage-phosphatase deficient PTEN mutant (PTEN-C124S) and phage-empty vector were purchased from OBiO Technology Company. The core vector and packing plasmids psPAX2 and the envelope plasmid pMD2.G were transfected into HEK293T cells using polyethylenimine. The medium was changed 6 h after transfection. Seventy-two hours post transfection, cell supernatants containing lentivirus were collected and passed through a 0.45 μm filter. GL261, U251 and GSC1 cells were introduced with lentivirus for 4 h. Forty-eight hours after introduction, the cells were selected with 2 µg/ml puromycin or 5 µg/ml blasticidin for 7-10 days to establish stably expressing cell lines. For animal models, GL261-shPTEN1 showed stable reduced expression of PTEN (GL261-shPTEN) and were selected to establish xenograft model. After establishing GL261-shPTEN1 stable cell lines collected with puromycin, we subsequently knocked down IL-12A in the GL261-shPTEN1 cell lines (GL261-shIL12) and collected cells with blasticidin.

**Chemicals**

The PI3K inhibitors GNE-317 were purchased from Selleck. Both chemicals are dissolved in DMSO to make a 10 mM stock solution for in vitro studies. The highest DMSO concentration (0.001% DMSO) used for in vitro studies is nontoxic to the cells. For *in vivo* studies, GNE-317 powder (20 mg/kg) is dissolved in 0.5% hydroxypropyl methylcellulose and 0.2% Tween-80 (Sigma Aldrich) to make a homogenous suspension and is administered by oral gavage.

**Animal Models**The study has been approved by the Animal Ethical and Welfare Committee of Sun Yat-sen University.

For the subcutaneous xenograft model, dissociated GSC1 cells (3 x 10^6^) in 100 µl of PBS were inoculated subcutaneously into the hind flanks of 4-week-old female BALB/c-nu/nu mice. When palpable tumors have developed (~100 mm^3^) in approximate 5 days, the mice were randomly divided into four groups. VSVΔ51 (3×10^7^ PFU/kg/day) was administered by tail vein injection on days 5-7. Tumor length and width were measured every 3 days, and the volume was calculated according to the formula (length×width^2^)/2.

The orthotopic intracranial xenograft model was established as previously described: Briefly, dissociated GL261-shPTEN cells (3 x 10^5^ cells) in 5 µl of PBS were implanted stereotactically into the striatum (2.2 mm lateral from the bregma and 2.5 mm deep) to generate intracranial tumors. Eight days after tumor implantation, the mice were randomly divided into groups and treated with different agents. PD-1 antibody (10 mg/kg/day) or isotype control antibodies (rat IgG2a) is administrated via intraperitoneal (i.p.) injection three times on the 8th, 10th and 12nd day. GNE-317 (20 mg/kg/day) is given by oral gavage (p.o.) for 5 consecutive days starting on day 8. VSVΔ51 (3×10^7^ PFU/kg/day) is injected via tail vein (i.v.) for 3 consecutive days on 8^th^ to 10^th^ day.

Rechallenge experiments: 5 long term survived mice after the triple-combination therapy of VSVΔ51, GNE-317 and anti-PD-1 were rechallenged on day 80^th^ with a twofold load of tumor cell GL261-shPTEN1 (6 x 10^5^) in the contralateral (left) flank. 5 Age-matched naïve mice were implanted the same amount of virus as control group.

Immune cell depletion experiments: Mice were administered via i.p. with anti-mouse CD8α (10 mg/kg), anti-mouse CD4 (10 mg/kg), or clodronate liposomes (first injection 50 mg/kg, followed by 25 mg/kg) on day 5, 7, 9, 11, 13 and 15. The control group received rat IgG2b isotype (i.p.) and empty liposomes (i.p*.*) with the same dose.

IL12 depletion study: Mice were administered via i.p. with anti-mouse IL12p70 (first injection 50 mg/kg, followed by 25 mg/kg) or isotype rat IgG on day 7 through 14.

*In vivo* tumor-derived IL-12 deletion study: Dissociated GL261-shPTEN1-shIL12 cells (3 x 10^5^ cells) in 5 µl of PBS were implanted stereotactically into the striatum to generate intracranial tumors. Mice were randomly divided into groups and treated with 1) PD-1 antibody (10 mg/kg/day) via i.p. three times on day 8th, 10th and 12nd; 2) GNE-317 (20 mg/kg/day) by oral gavage for 5 consecutive days starting on day 8^th^; 3) VSVΔ51 (3×10^7^ PFU/kg/day) is injected via tail vein (i.v.) for 3 consecutive days on 8^th^ to 10^th^ day.

**Mouse Bioluminescence Imaging**
Mice implanted with GBM cells expressing luciferase were injected intraperitoneally with a luciferin solution (15 mg/mL in DPBS, dose of 150 mg/kg). The bioluminescence images were acquired using the IVIS Lumina system and analyzed by Living Image software. Imaging experiments were conducted at the Animal Imaging platform of the Experimental Animal Center of Sun Yat-Sen University.

**Quantitative RT-PCR**
Total RNA was extracted with TRIzol reagent (Invitrogen). A reverse transcription system (Promega) was used to synthesize cDNA. SuperReal PreMix SYBR Green (Tiangen) and an ABI Q5 Detection System were used for qRT-PCR. The mRNA results were normalized to GAPDH expression. Primers for qRT-PCR are listed in Supplementary information, Table S1.

**Flow Cytometry Analysis**

Annexin-V expression was detected in U251 cells and U251-PTEN cells after culture for 24 h with or without the combination treatment of GNE317 (1 µM) and VSVΔ51 (0.01 MOI). The cells were centrifuged, counted, resuspended in FACS buffer (2% inactivated fetal calf serum in PBS), incubated with Annexin V-APC and PI (MultiSciences) for 15 min, washed and resuspended in FACS buffer, and then analyzed using Cytoflex (Beckman Coulter) and CytExpert.

For multicolor flow cytometric analysis, brain tumor quadrants were harvested, minced, incubated with a Brain Tumor Dissociation Kit (Miltenyi, 130-095-942), triturated, passed through a 70 mm screen, resuspended in FACS buffer, and stained with fluorochrome-conjugated anti-mouse antibodies from BioLegend or eBioscience, as well as appropriate isotype control antibodies. A Zombie Red Fixable viability kit (BioLegend) was used to stain dead cells. We followed a ‘no-wash’ sequential staining protocol (BioLegend) to stain dead cells and for surface staining. Intracellular FoxP3 staining was performed following the FoxP3 intracellular staining protocol (BioLegend). For single-color compensation controls, UltraComp eBeads (eBioscience) were used and stained with each of eight fluorescently conjugated antibodies according to the manufacturer’s instructions. For the Zombie Red assay, cells from the nontumor and tumor quadrants, respectively, were used as single color compensation controls. All samples were run in a Cytoflex flow cytometer. Data were analyzed with CytExpert software. Technicians acquiring and gating the data were blinded to the treatments.Detailed information about the antibodies used is listed in Supplementary information, Table S2.

**Tissue virus titration**Virus titer were measured as previously described.[^2^](#_ENREF_2) GSC1-shNC or GSC1-shPTEN1 cells (3×10^6^ cells/mouse) were inoculated subcutaneously into the hind flank of 4-week-old female BALB/c-nu/nu mice. After 8 days, palpable tumors developed (~300 mm3), VSVΔ51 (3×10^7^ pfu) was intravenously injected, and tumors were resected at 24 h after infection. Tumor tissues were homogenized using gentleMACS Dissociator (Miltenyi Biotec). Briefly, tumors were weighed and collected in 2 ml of ice-cold 1×PBS and then transferred to gentleMACS M Tube. Tubes were attached upside down on the sleeve of the dissociator, and gentleMACS Program RNA_1 was initiated to collect the homogenate. Homogenate was centrifuged at 600×g for 10 min, and supernatant was collected and titered on BHK-21 cells to quantify infectious virus by the TICD50 method.

**Dual-luciferase reporter assay**
U251 or GL261 cells were seeded in 24-well plates and then transfected with IFN-β-luc together with Renilla luciferase as a control. Twenty-four hours after transfection, the cells were stimulated with VSV for another 12 h. The cells were lysed with passive lysis buffer and subjected to measurements of dual-luciferase activity with a Luciferase Reporter Assay System (Promega). The lysates left were collected for western blot analysis.

**Immunoblot analysis**

For immunoblot analysis, whole-cell extracts were collected and lysed in RIPA lysis buffer containing 50 mM Tris-HCl, pH 8.0, 150 mM NaCl, 1.0% (v/v) Triton X-100, 1.0% sodium deoxycholate and 0.1% SDS. The lysates were subjected to SDS-PAGE, transferred onto PVDF membranes and then blotted with the indicated antibodies. Detailed information about the antibodies used is listed in Supplementary information, Table S2.

**Immunohistochemistry and Immunofluorescence**For immunohistochemistry, the expression of PTEN, CD3 and IL-12 in tumors was assessed by immunohistochemistry. Briefly, tumor sections (4 μm) were dewaxed in xylene, hydrated in decreasing concentrations of ethanol, immersed in 0.3% H_2_O_2_-methanol for 30 min, washed with phosphate-buffered saline, and probed with monoclonal antibodies or isotype control at 4°C overnight. After being washed, the sections were incubated with biotinylated goat anti-rabbit or anti-mouse IgG at room temperature for 2 h. Immunostaining was visualized with streptavidin/peroxidase complex and diaminobenzidine, and sections were then counterstained with hematoxylin. For immunofluorescence, tumors were harvested and postfixed overnight in periodate-lysine-paraformaldehyde fixative at 4°C. Sections were cut and rehydrated in PBS, permeabilized in 0.1% Triton X-100, blocked in 5% goat serum and incubated with primary antibodies including GFP (reporter gene of VSVΔ51), PTEN and cleaved caspase3 overnight at 4°C. After the slides were washed in PBS, they were incubated with secondary antibodies, counterstained with DAPI (Invitrogen), washed in 0.1% Triton X-100 and mounted using Prolong Diamond antifade (Invitrogen). Detailed information about the antibodies used is listed in Supplementary information, Table S2.

**Cell viability assay**

The cell viability of the adherent GL261 and U251 cells was measured by 3-(4,5-dimethylthiazol-2-yl)-2,5-diphenyltetrazolium bromide (MTT) assay. Cells were seeded in 96-well plates at 3,000 cells per well in 0.1 ml medium. MTT was added to the cells (1 mg/ml final concentration), and the cells were allowed to grow at 37°C for another 3 h. MTT-containing medium was removed, and the MTT precipitate was dissolved in 100 μl of DMSO. The optical absorbance was determined at 490 nm using a microplate reader (Bio-Rad).

The cell viability of the suspending GSC1 cells was measured by Cell Counting Kit-8 (CCK-8). Cells were seeded in 24-well plates at 2,0000 cells per well in 0.5 ml medium. 50 ul of CCK-8 was added to cells, and then cells were allowed to grow at 37°C for another 3 h. CCK-8 containing medium was collected, then the optical absorbance was determined at 450 nm using a microplate reader (Bio-Rad).

**ELISA**
The production and secretion of human IFN-β in U251 supernatants and mouse ifn-β in GL261 cells were measured with a human IFN-β ELISA kit (R&D Systems) and mouse IFN-β ELISA kits (R&D Systems), respectively. The IL-12p70 ELISA kit was from PeproTech and was used for the detection of proteins in U251 cell supernatants.

**Cytokines analysis by luminex technology**

After U251 cells were adherent, cells were washed with PBS for twice and cultured with [fetal bovine serum](https://www.sciencedirect.com/topics/neuroscience/fetal-bovine-serum) free DMEM. Then cells were treated with GNE317 (1 µM) and VSVΔ51 (0.01 MOI) for 24 h. The conditioned medium were collected and sent to Wayen Biotechnologies (shanghai) for cytokine screening using Bio-Plex Pro Human 48-plex Panel (Biorad，#12007283).

**Statistical analysis**
All experiments were repeated at least two times, and the data are shown as the mean ± SD. Statistical significance between different groups was calculated by two-tailed unpaired Student’s t-test or two-way ANOVA. For the survival curve of mice, the log-rank (Mantel-Cox) test was used for the comparison. A *p* value < 0.05 was considered to be significant. Data were analyzed with GraphPad Prism 7.0 Software.

***Reference***

1 Xing, F. *et al.* The Anti-Warburg Effect Elicited by the cAMP-PGC1α Pathway Drives Differentiation of Glioblastoma Cells into Astrocytes. *Cell Rep* **18**, 468-481, doi:10.1016/j.celrep.2016.12.037 (2017).

2 Zhang, H. *et al.* Targeting VCP enhances anticancer activity of oncolytic virus M1 in hepatocellular carcinoma. *Sci Transl Med* **9**, doi:10.1126/scitranslmed.aam7996 (2017).


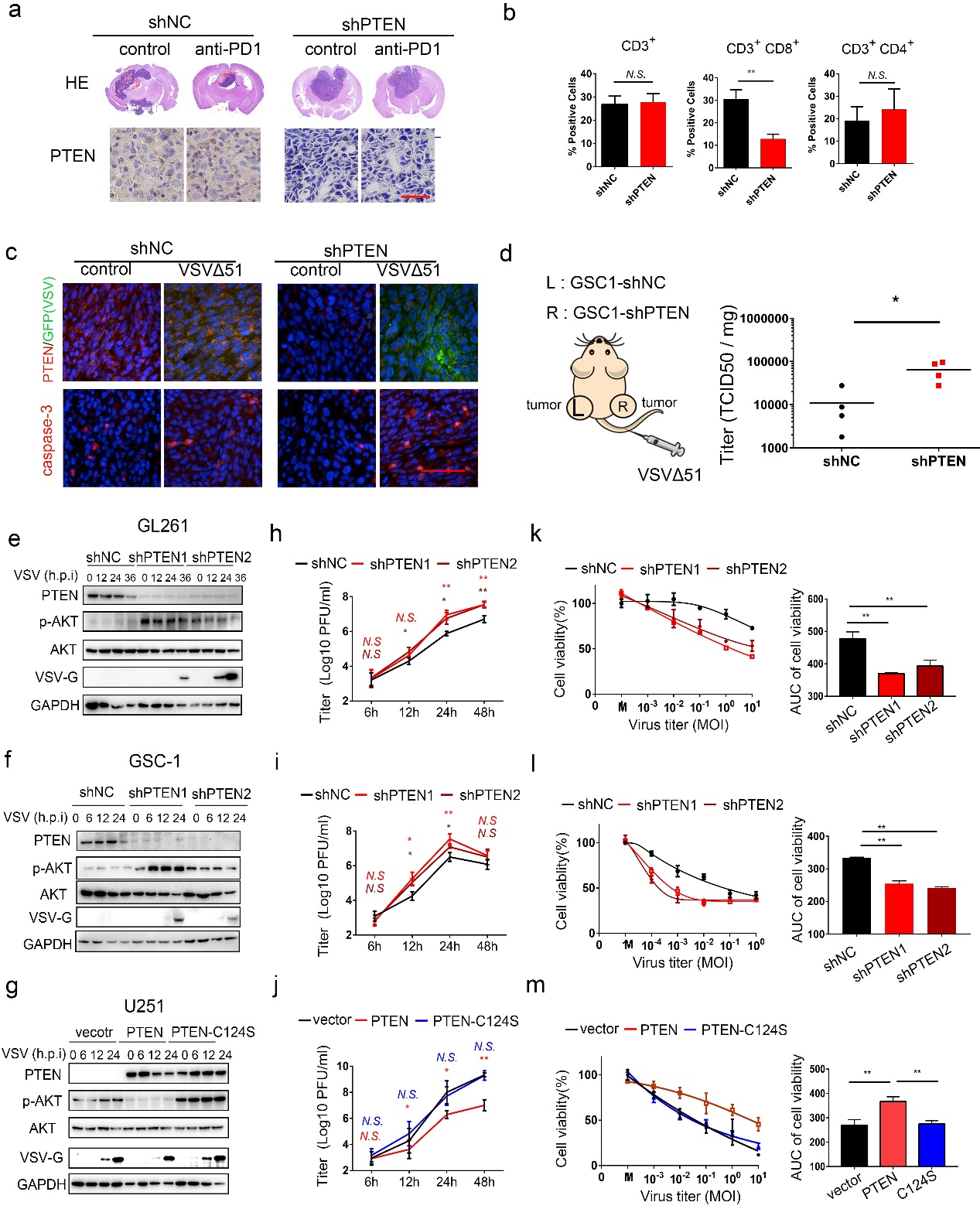


**Fig. S1** The differential response of PTEN deficiency GBM to anti-PD-1 therapy and OV treatment.

**a, b** Knockdown of PTEN led to resistance to anti-PD-1 therapy. **a** C57 mice were implanted with 3*10^5^ GL261-shNC or GL261-shPTEN cells on day 0. Then, the mice were treated with anti-PD-1 antibody (10 mg/kg) at day 8th, 10th and 12nd. Fourteen days after inoculation, the mice were euthanized. The brain tumors were sectioned to detect tumor growth by H&E staining (scale bar = 1 cm) and to evaluate the expression of PTEN by immunohistochemistry (scale bar = 50 μm). **b** The percentages of CD3^+^ cells and CD3^+^ sorted CD4^+^ and CD8^+^ subsets in the tumors were analyzed by Flow Cytometry.

**c** Knockdown of PTEN increased the sensitivity to OV *in vivo*. Nude mice were implanted with patient derived glioma stem cell-1 (GSC-1) to develop subcutaneous xenograft. 3*10^6^ GSC1-shNC or GSC1-shPTEN cells were implanted on day 0. Then, the mice were treated with 3*10^7^ PFU VSV on day 5 through 7. Eight days after inoculation, mice were euthanized and subjected to [immunofluorescence](http://www.baidu.com/link?url=8fKR_md0k2inzur3tWJ4eVD2MMHjdMlukN7oHujhqC18S8GNRfAqw7k-jOT9JUQz9RUI0rNch5CHroNdFz5s2UljGulzoUy3WnkXIyj3Te_uAFt-f1k01_07z-qILGD2) to evaluate the expression of GFP (reporter gene for VSVΔ51), PTEN and cleaved caspase-3, scale bar = 50 μm.

**d** Knockdown of PTEN enhanced the tropism and replication of OV in a bilateral tumor model. The bilaterally implanted mice were treated with 3*10^7^ PFU VSV by intravenous injection. Subcutaneous tumor titers are shown for animals sacrificed 24 h after virus administration (n = 4 per group). Virus production in tumor tissue was measured by the TCID50 method.

**e-g** Immunoblot analysis of PTEN, AKT, phosphorylation of AKT and viral protein (VSV-G) in GL261(**e**), GSC-1(**f**) and U251(**g**). GL261 and GSC-1 cells were transfected with shNC, shPTEN1 or shPTEN1; U251 cells (PTEN loss) were overexpressed with vector, PTEN or the phosphatase-deficient PTEN mutant (PTEN-C124S). VSVΔ51 MOI=0.1 for GL261 and U251 cells; VSVΔ51 MOI=0.01 for GSC-1 cells. h.p.i means hours post infection.

**h-j** The kinetics of virus growth were measured in GL261(**h**), GSC-1(**i**) and U251(**j**) . Virus production in the medium was measured by the TCID50 method for the indicated time points.

**k-m** The oncolysis effect of OV was detected in GL261(**k**), GSC-1(**l**) and U251(**m**) . Left panel: Cell were infected with VSVΔ51 at indicated MOI. Cell viability was measured by MTT assays after 48 h treatment. Right panel: The area under curve (AUC) of cell viability were calculated.

The mean ± SD is shown. **, p < 0.01; *, p < 0.05; *N.S.*, no significant differences.


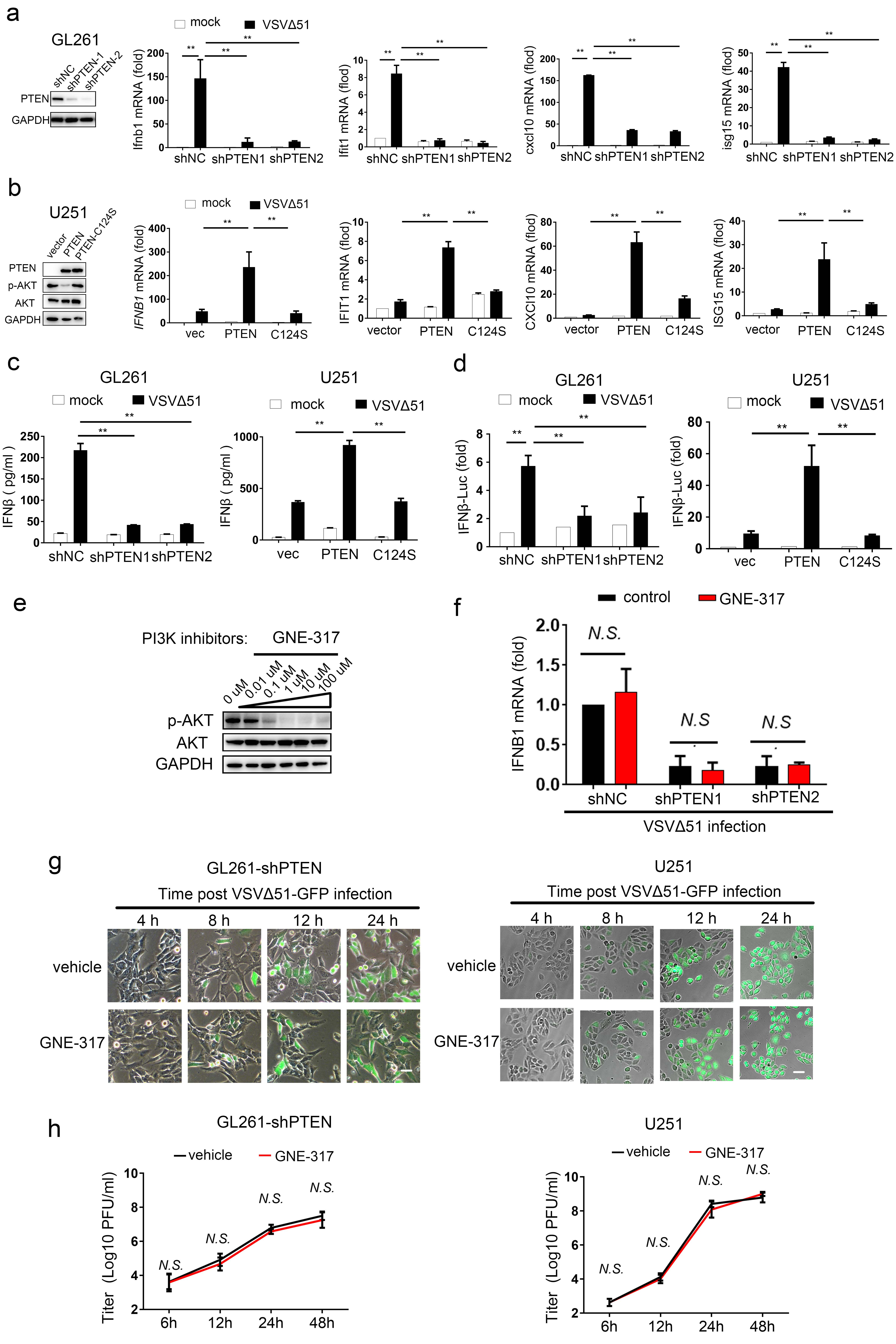


**Fig. S2** PTEN deficiency blunted the type I interferon pathway and anti-virus response via PI3K independent way*.*

**a-b** PTEN regulated the expression of anti-virus related genes. GL261 cells (PTEN intact) were transfected with shNC, shPTEN1 or shPTEN1; U251 cells (PTEN loss) were overexpressed with vector, PTEN or the phosphatase-deficient PTEN mutant (PTEN-C124S).The qRT-PCR analysis of the mRNA levels of ifnb1 and ISGs (ifit1, cxcl10 and isg15) in GL261 cells (**a**) and U251 cells (**b**). The cells were stimulated with VSVΔ51 for 8 h, MOI=0.1 (n = 3).

**c** ELISA analysis of IFN-β production in GL261 cells and U251 cells. The cells were stimulated with VSVΔ51 for 12 h, MOI=0.1.

**d** Dual-luciferase reporter assays were performed in GL261 cells and U251 cells. Cells were transfected with a luciferase reporter plasmid containing IFN-β for 24 h and then left uninfected or infected with VSVΔ51 for 8 h; luciferase reporter activity was normalized to that of Renilla luciferase (n = 3).

**e-f** PI3K inhibitors GNE317 could not rescue the inhibition of *Ifnb1* expression caused by PTEN knockdown in GL261 cells. **e** Immunoblot analysis of AKT and AKT phosphorylation in GL261-shPTEN cells. Cells were treated with the PI3K inhibitors GNE317 at the indicated concentrations for 12 h. **f** qRT-PCR analysis of the mRNA levels of *Ifnb1* in GL261 cells. Cells were treated with 1 µM GNE-317 and infected with VSVΔ51 (MOI=0.1) for 12 h.

**g-h** PI3K inhibitor GNE-317 did not alter infectivity and viral proliferation of VSVΔ51. **g** Phase-contrast and fluorescence microscopy images were captured to evaluate virus infection in GL261-shPTEN cells and U251 cells. Scale bar = 20 μm. **h** Virus titers were measured by TCID50 in GL261-shPTEN cells and U251 cells. Cells were treated with GNE-317 (1 µM) and infected with VSVΔ51 for the indicated times.

The mean ± SD is shown. **, p < 0.01; *, p < 0.05; *N.S.*, no significant differences.


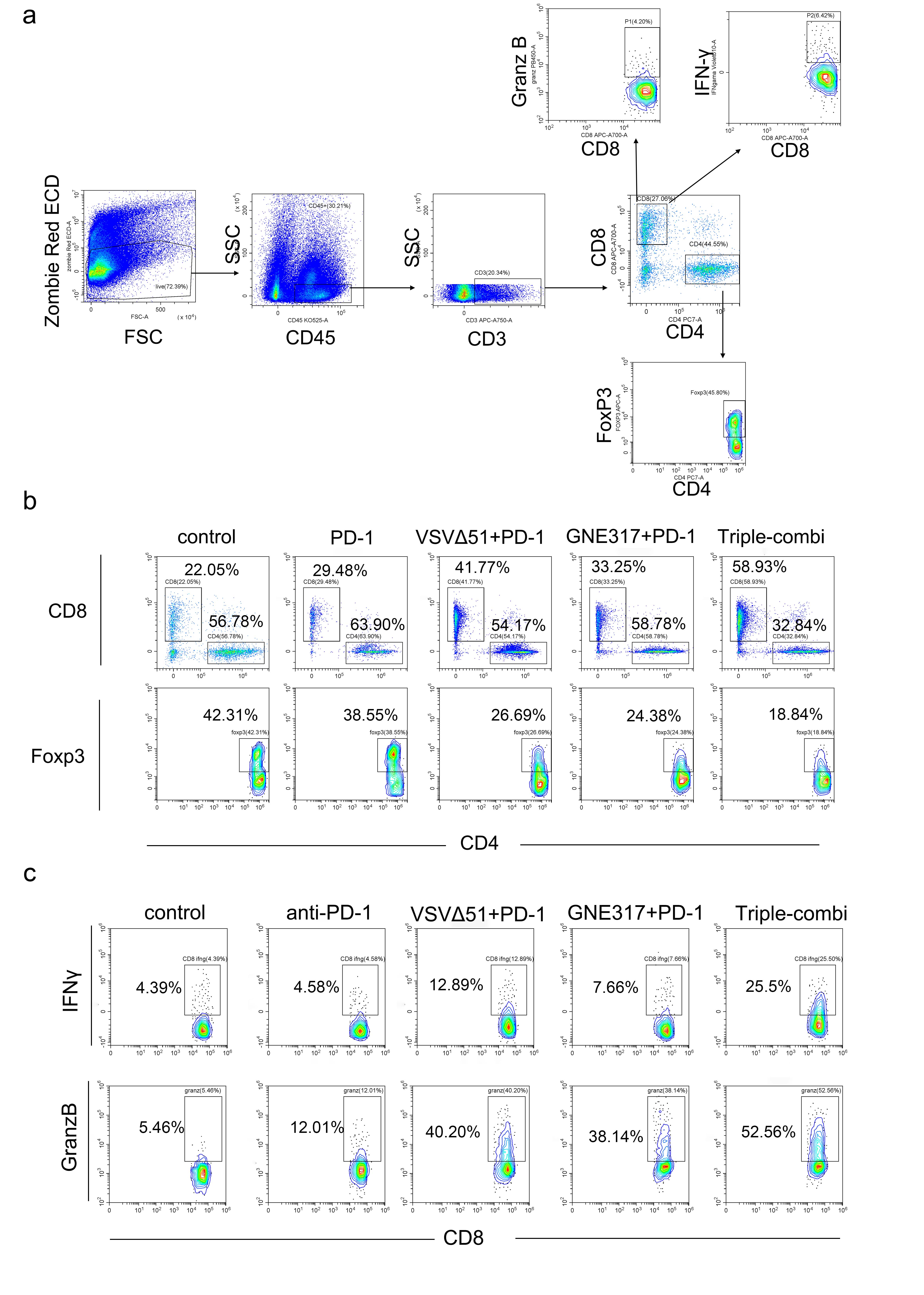


**Fig. S3** Depiction of the gating strategy employed for flow cytometry and the effect of the triple combination on immune cell infiltration and function.

**a** Gating strategy and representative flow cytometry plots for the assessment of CD4^+^, CD8^+^ T cells and Tregs (Foxp 3^+^) in control tumors. The function of CD8^+^ T cells was further evaluated by IFNγ and granzyme B expression.

**b** Representative flow cytometry plots for the assessment of CD4^+^, CD8^+^ T cells and Tregs (Foxp 3^+^).

**c** Representative flow cytometry plots for the assessment of the function of CD8^+^ T cells by measuring IFNγ and granzyme B.


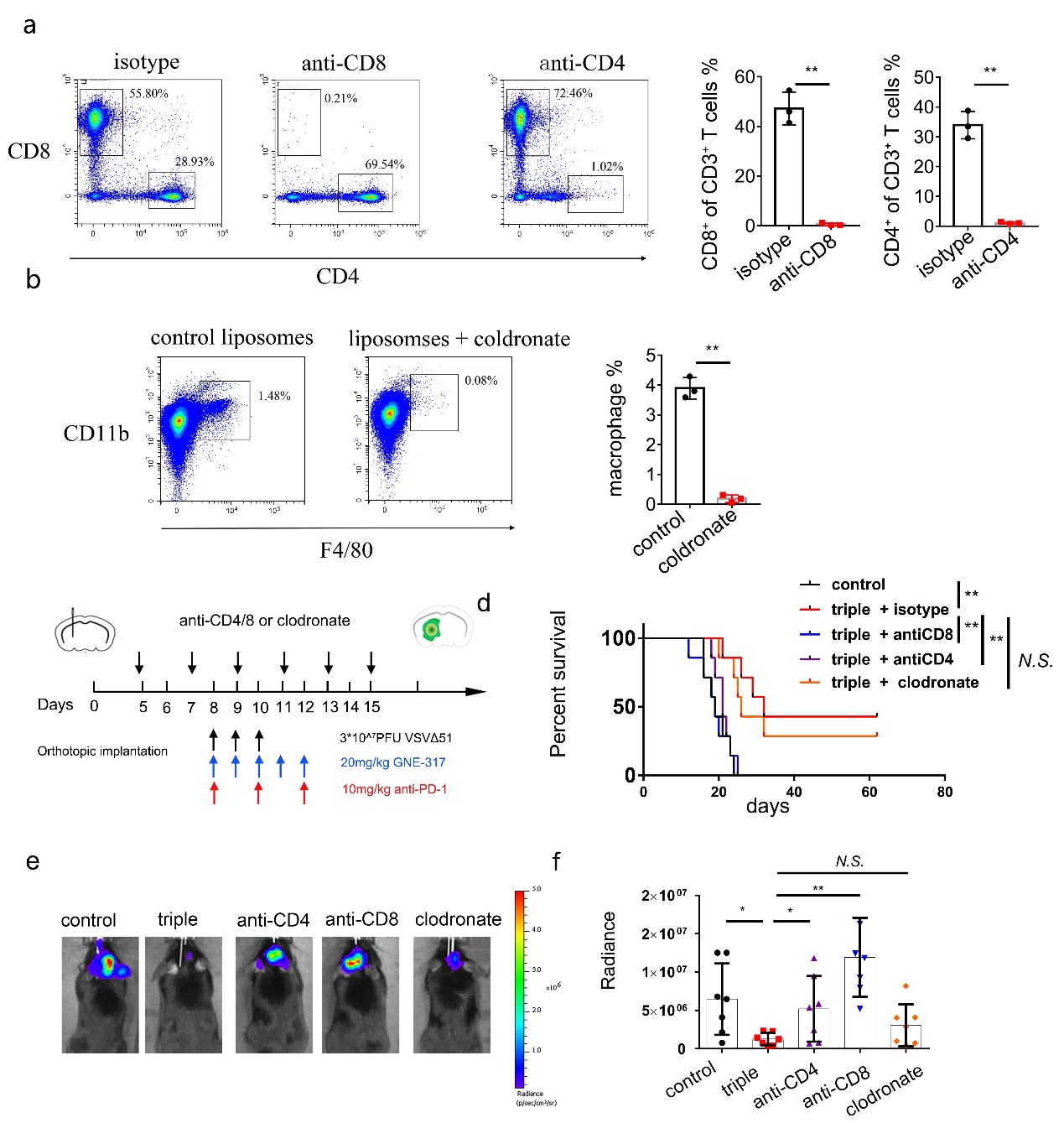


**Fig. S4** Therapeutic effect of triple combination depended on the CD4^+^ and CD8^+^ T cells, but not macrophages.

**a-b** Validation of immune cell depletion. In vivo effects of depleting antibodies (CD4 and CD8) on CD4^+^ and CD8^+^ T cell populations (**a**) and clodronate liposomes on macrophage (CD11b^+^, F4/80^+^) populations (**b**) in splenocytes from treated mice. C57 mice were injected with either anti-mouse CD8 (10 mg/kg), anti-mouse CD4 (10 mg/kg), control liposomes or clodronate liposomes (first dose 50 mg/kg followed by 25 mg/kg) by intraperitoneal (i.p.) injection every 72 h for 3 doses. After the last dose, splenocytes were isolated and stained with anti-mouse CD4, CD8, CD11b and F4/80 antibodies and then analyzed by flow cytometry.

**c** The timeline and does of treatment in depletion experiments.

**d** Kaplan-Meier survival curve of the mice bearing GL261-shPTEN1 tumors. The median survival of the mice was determined as follows: control group, 19 days; triple-combination + isotype antibody group, 32 days; triple-combination + anti-CD8 group, 19 days; triple-combination + anti-CD8 group, 21 days; triple-combination + clodronate group, 26 days.

**e** Tumor progression was monitored via bioluminescence imaging of luciferase activity in the GL261-shPTEN xenograft mice on day 14 (n = 6).

**f** Quantitative radiance of mice on day 14 was analyzed as shown on the right graph.

The mean ± SD is shown. **, p < 0.01; *, p < 0.05; *N.S.*, no significant differences.


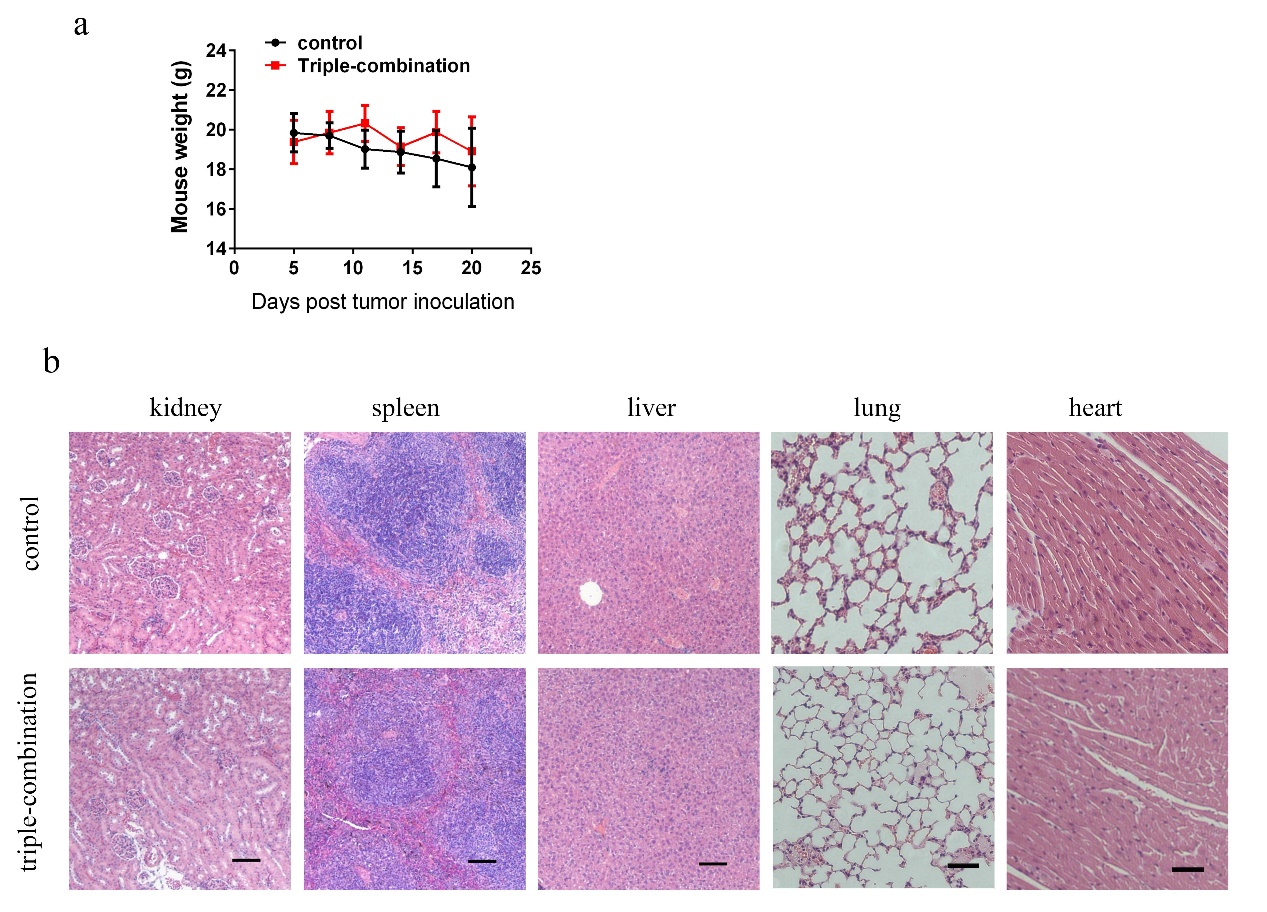


**Fig. S5** Safety and potential toxicity evaluation of the triple combination in immunocompetent mice.

**a** Body weight was measured every 3 d. Immunocompetent C57 mice received triple combinations of GNE317, VSVΔ51 and anti-PD-1 or vehicle. The mean ± SD is shown (n = 8).

**b** H&E staining of vital tissues, including kidney, spleen, liver, lung and heart. (Scale bars: 100 μm.).


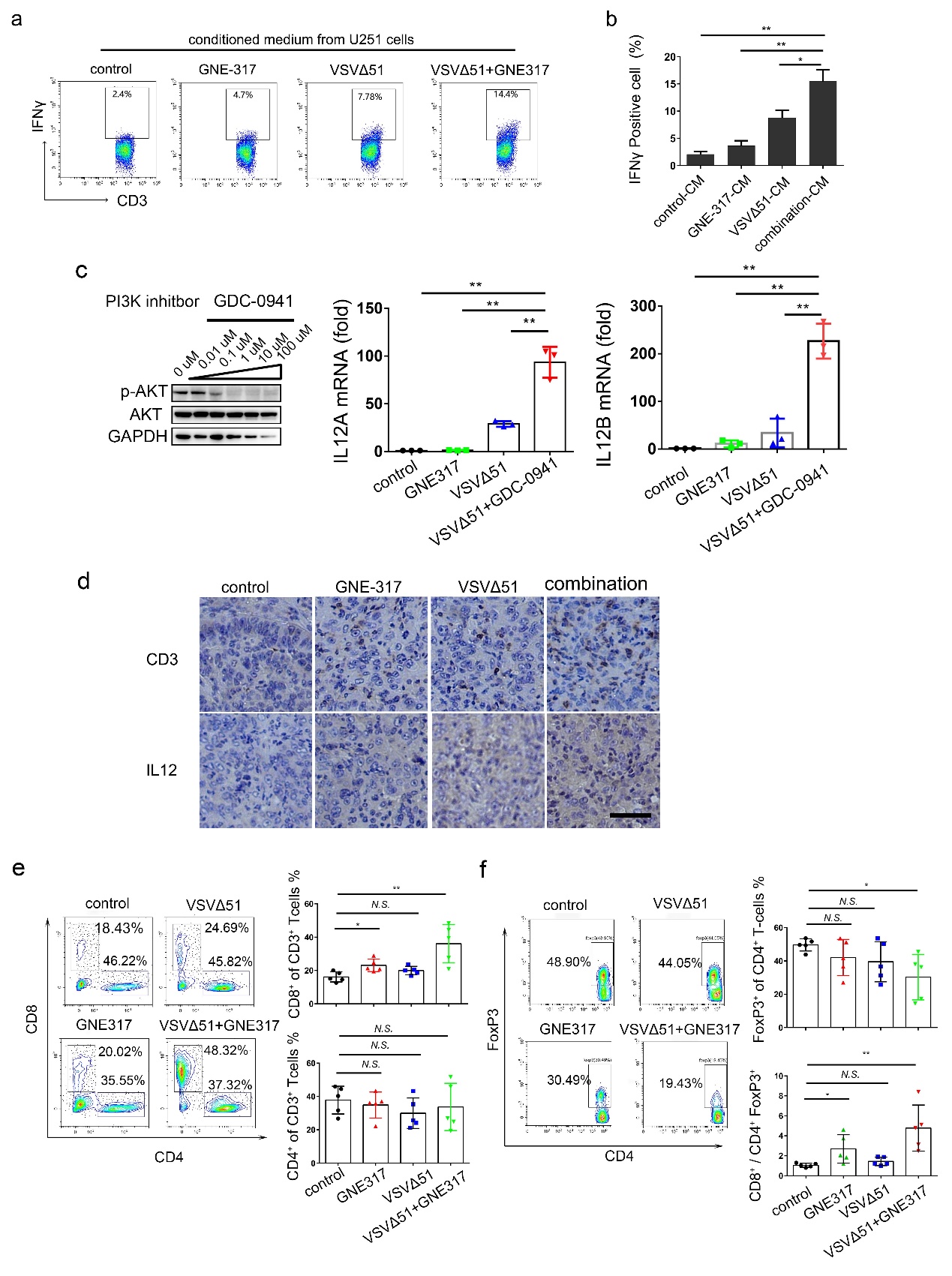


**Fig. S6** Combination oncolytic virus and GNE-317 induced IL-12 expression and reshaped the immune microenvironment.

**a-b** Conditioned medium from treated glioma cells activated IFNγ expression of Jurkat T cell. Conditioned medium form U251 cells was collected upon treatment with control, GNE317 (1 uM), VSVΔ51(MOI=0.1) and the combination for 24 h. The conditioned medium was then inactivated by UV light to eliminate the effect of the virus. Then Jurkat T cells were induced by conditioned medium for 24 h. **a** Flow cytometry analysis of IFNγ positive Jurkat T cells was conducted. **b** Quantitative analysis of percentage of IFNγ positive Jurkat T cells.

**c** The combination of other PI3K inhibitor GDC-0941 and VSVΔ51 could also induced the expression of *IL12A* and *IL12B* expression U251 cells. Right graph: Immunoblot analysis of AKT and AKT phosphorylation in U251 cells. Cells were treated with the PI3K inhibitors GNE317 at the indicated concentrations for 12 h. Left graph: qRT-PCR analysis of the mRNA levels of *IL12A* and *IL12B*. Cells were treated with 1 µM GDC-0941 and infected with VSVΔ51 (MOI=0.1) for 12 h.

**d** Immunohistochemistry detection of CD3 and IL-12 in brain tumor tissue. Mice were intracranially transplanted with GL261-shPTEN1 cell and treated with 3*10^7^ PFU VSVΔ51 and 20 mg/kg GNE-317 on day 6 through 8. Mice were euthanized on day 14. Scale bar = 50 μm.

**e** Flow cytometry analysis of the percentages of CD4^+^ T cells and CD8^+^ T cells among CD45^+^ cells in brain tumor tissue. Mice were treated the same as **c**.

**f** Flow cytometry analysis of the percentage of Treg cells (Foxp3^+^) among CD4^+^ T cells in brain tumor tissue. Mice were treated the same as **c**.

**, p < 0.01; *, P < 0.05; *N.S.*, no significant differences.


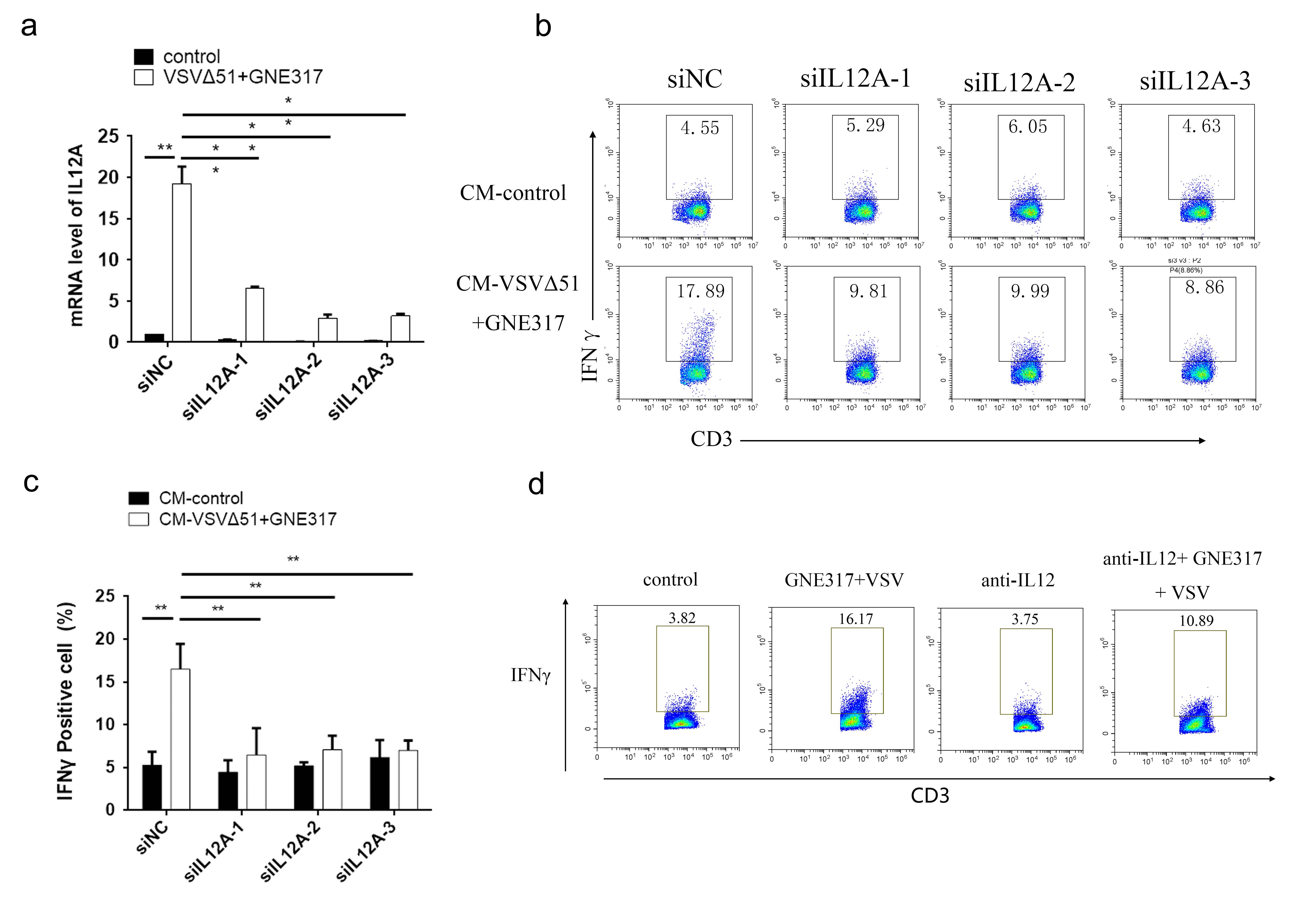


**Fig. S7** Glioma-derived IL-12 mediated T cell activation *in vitro*.

**a** Knockdown IL12A of U251 cells decrease the conditioned medium induced IFNγ-expression in Jurkat T cells. qRT-PCR analysis of the mRNA levels of IL12A in U251 cells. U251 cells were transfected with siNC, siIL12A-1, siIL12A-2 and siIL12A-3 for 24 h and then treated with VSVΔ51 and GNE-317 for 24 h.

**b** Jurkat T cells were induced by conditioned medium for 24 h. Then flow cytometry analysis of percentage of IFNγ-positive cells was conducted.

**c** Quantitative analysis of percentage of IFNγ positive cells.

**d** The neutralizing antibody to IL-12 blocked the conditioned medium induced IFNγ expression in Jurkat T cells. The conditioned medium was pretreated with anti-IL-12 antibody for depletion or not. Flow cytometry analysis of the percentage of IFNγ-positive Jurkat T cells was conducted.

The mean ± SD is shown. **, p < 0.01; *, p < 0.05; *N.S.*, no significant differences.


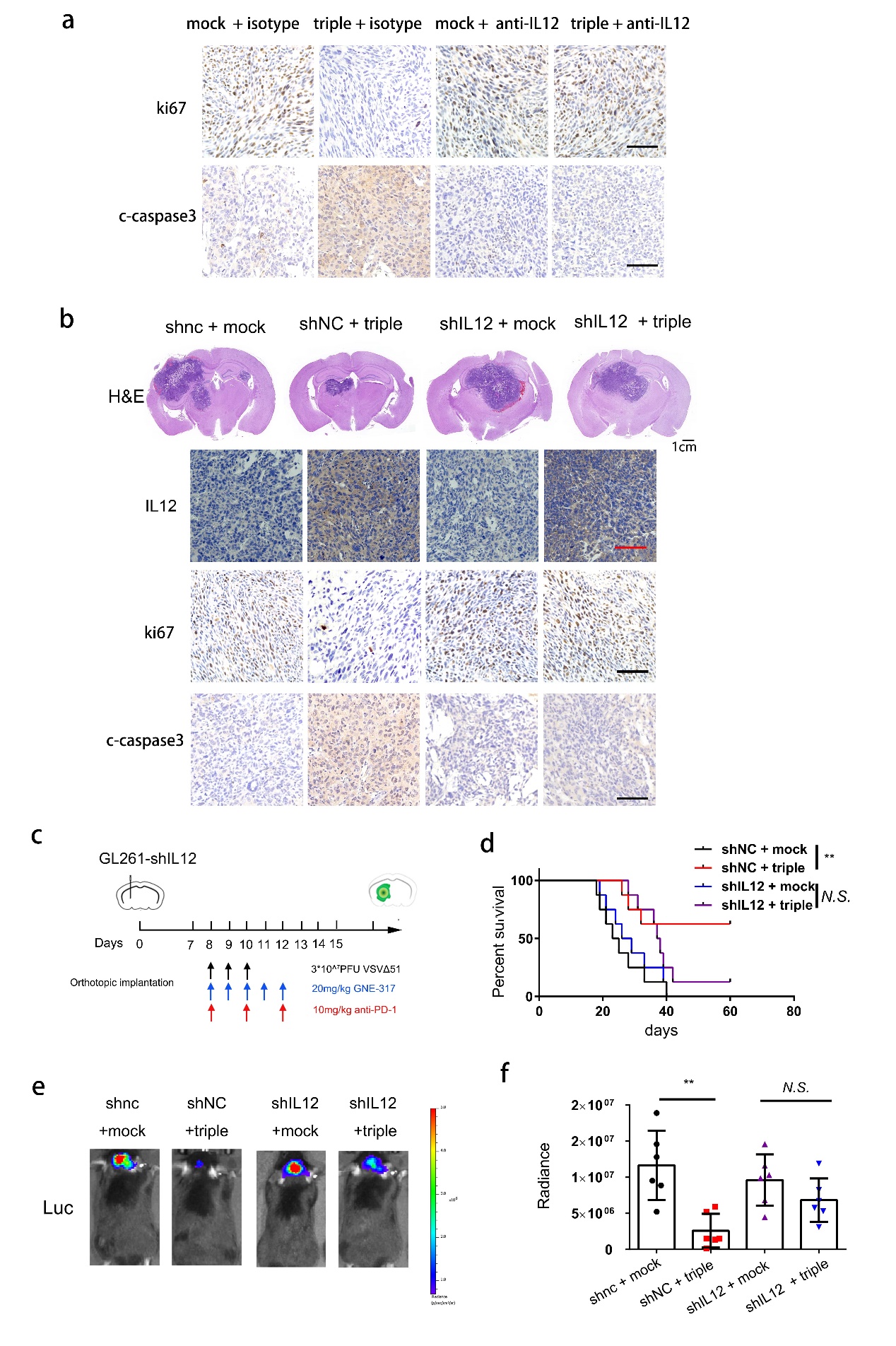


**Fig. S8** Knockdown of IL-12 in glioma dampened the therapeutic effect of the triple combination.

**a** Depletion antibody against IL-12 was injected to reduce the glioma derived IL-12. The mice were euthanized and subjected to immunohistochemistry (scale bar = 100 μm) to evaluate the expression of Ki67 and cleaved caspase-3 on fifteen days after tumor inoculation.

**b** GL261 cells were transfected with shNC or shIL12, then intracranially transplanted into C57 mice. Fifteen days after inoculation, the mice were euthanized and subjected to H&E staining to detect tumor growth and immunohistochemistry (scale bar = 100 μm) to evaluate the expression of IL-12, Ki67 and cleaved caspase-3.

**c** The timeline of IL-12 depletion experiments.

**d** Kaplan-Meier survival curve of mice bearing GL261 tumors. The median survival of the mice was determined as follows: shNC + mock group, 24 days; shNC + triple group, over half of the mice survived; shIL12 + mock group, 27.5 days; shIL12 + triple group, 37.5 days.

**e** Monitoring of tumor progression via bioluminescence imaging of luciferase activity in GL261-shPTEN xenograft mice on day 14 (n = 6).

**f** Quantitative radiance of mice was analyzed. **, p < 0.01; *, P < 0.05; *N.S.*, no significant differences.


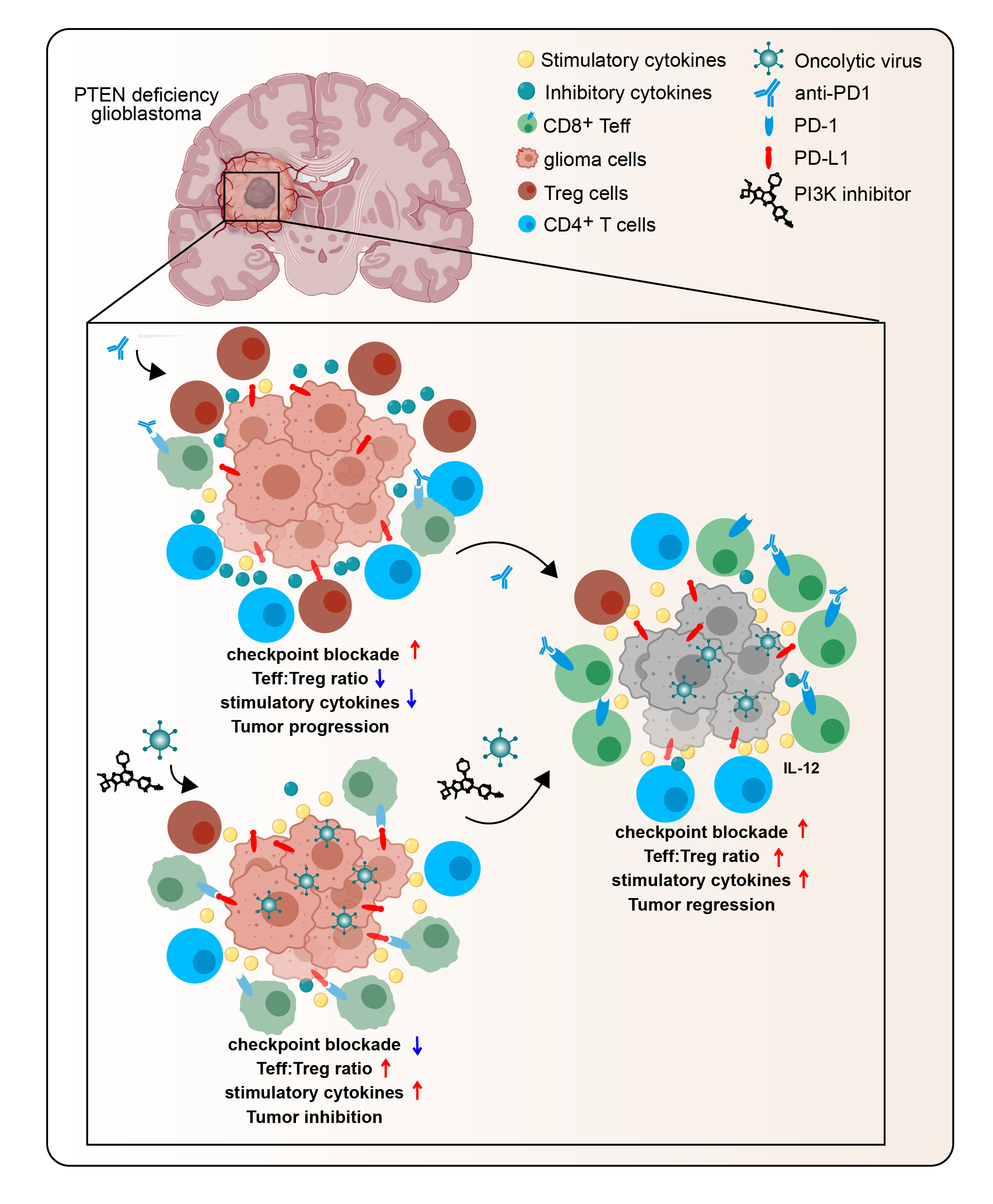


**Fig. S9** Graphical depiction of the modulation of the tumor microenvironment induced by OV, PI3K inhibitor and ICB combination therapy.

In GBM, PTEN deficiency lead to decreased CD8^+^ Teff cell infiltration resulting in an immunosuppressive microenvironment. Treatment with a single anti-PD-1 agent will block immune checkpoint interaction. However, bare infiltration of Teff cells results in no response to ICB. Treatment with the combination of OV and PI3K inhibitor will enhance the Teff: Treg ratio and induces the tumor derived IL-12 expression, leading to the reshaping of the immune state. While the increased infiltration Teff cells will be inhibited by immune checkpoint PD-1 and PD-L1 interaction. With triple combination therapy, the activation of increased Teff cell is licensed by checkpoint blockade, triggering the anti-tumor immune response and the tumor regression.

Supplementary information, Table S1 Primers for qRT-PCR

|  | Species | Primer （5'-3'） |
| --- | --- | --- |
| *GAPDH* Forward | Human | ATGACATCAAGAAGGTGGTG |
| *GAPDH* Reverse | Human | CATACCAGGAAATGAGCTTG |
| *IFNB1* Forward | Human | AGGACAGGATGAACTTTGAC |
| *IFNB1* Reverse | Human | TGATAGACATTAGCCAGGAG |
| *IFIT1* Forward | Human | CCTCCTTGGGTTCGTCTACA |
| *IFIT1* Reverse | Human | GGCTGATATCTGGGTGCCTA |
| *ISG15* Forward | Human | GAGAGGCAGCGAACTCATCTT |
| *ISG15* Reverse | Human | CCAGCATCTTCACCGTCAGG |
| *CXCL10* Forward | Human | GCTCTACTGAGGTGCTATGTTC |
| *CXCL10* Reverse | Human | GGAGGATGGCAGTGGAAGTC |
| *IL12B* Forward | Human | GCGGAGCTGCTACACTCTC |
| *IL12B* Reverse | Human | CCATGACCTCAATGGGCAGAC |
| *IL12A* Forward | Human | CCTTGCACTTCTGAAGAGATTGA |
| *IL12A* Reverse | Human | ACAGGGCCATCATAAAAGAGGT |
| *Gapdh* Forward | Mouse | ATGGTGAAGGTCGGTGTGAA |
| *Gapdh* Reverse | Mouse | CGCTCCTGGAAGATGGTGAT |
| *Ifnb1* Forward | Mouse | CCGAGCAGAGATCTTCAGGAA |
| *Ifnb1* Reverse | Mouse | CCTGCAACCACCACTCATTCT |
| *Isg15* Forward | Mouse | CCTCTGAGCATCCTGGTGAG |
| *Isg15* Reverse | Mouse | ACTGGTCTTCGTGGACTTGTT |
| *Ifit1* Forward | Mouse | CCAAGTGTTCCAATGCTCCT |
| *Ifit1* Reverse | Mouse | GGATGGAATTGCCTGCTAGA |
| *Cxcl10* Forward | Mouse | TCAGGCTCGTCAGTTCTAAGTT |
| *Cxcl10* Reverse | Mouse | GATGGTGGTTAAGTTCGTGCTT |
| *Il12a* Forward | Mouse | TGCCTTGGTAGCATCTATGAGG |
| *Il12a* Reverse | Mouse | CGCAGAGTCTCGCCATTATGAT |
| *Il12b* Forward | Mouse | GTCCTCAGAAGCTAACCATCTCC |
| *Il12b* Reverse | Mouse | CCAGAGCCTATGACTCCATGTC |

Supplementary information, Table S2 Detailed information of antibodies, drugs and kits used in this research

| Antibodies | source | Catalog # |
| --- | --- | --- |
| Phospho-Akt (Ser473) | CST | 9271S |
| Akt | CST | 9272S |
| PTEN | CST | 9188S |
| β-tubulin | Proteintech | 10094-1-AP |
| GAPDH | Proteintech | 60004-1-Ig |
| GFP | Proteintech | 50430-2-AP |
| VSV-G [8G5F11] | kerafast | EB0010 |
| Zombie Red Fixable | Biolegend | 423109 |
| CD3 Monoclonal Antibody (OKT3), FITC, eBioscience™ | Invitrogen | 11-0037-42 |
| Granzyme B Monoclonal Antibody (GB11), PE, eBioscience™ | Invitrogen | 12-8899-41 |
| IFN gamma Monoclonal Antibody (4S.B3), PE, eBioscience™ | Invitrogen | 12-7319-42 |
| CD3 Monoclonal Antibody (UCHT1), Alexa Fluor 700, eBioscience™ | Invitrogen | 56-0038-41 |
| Alexa Fluor® 647 anti-human FOXP3 | Biolegend | 320114 |
| PE anti-mouse CD274 (B7-H1, PD-L1) | Biolegend | 124308 |
| APC/Cyanine7 anti-mouse CD3ε | Biolegend | 100330 |
| Alexa Fluor® 700 anti-mouse CD8a | Biolegend | 100730 |
| PE/Cyanine7 anti-mouse CD4 | Biolegend | 100528 |
| Brilliant Violet 605™ anti-mouse IFN-γ | Biolegend | 505840 |
| InVivoMab anti-mouse PD-1 | Bioxcell | BE0146 |
| InVivoMAb rat IgG2a isotype control | Bioxcell | BE0089 |
| InVivoMab anti-mouse CD8α | Bioxcell | BE0061 |
| InVivoMab anti-mouse CD4 | Bioxcell | BE0003 |
| InVivoMab anti-mouse IL-12 p75 | Bioxcell | BE0233 |
| InVivoMab rat IgG2b isotype control | Bioxcell | BE0090 |
| InVivoMab anti-human IL-12 p70 | Bioxcell | BE0234 |
| IL-12 p35 Polyclonal | Invitrogen | PA5-79460 |
| CD3 | servicebio | GB13014 |
| Cleaved-Caspase3 | servicebio | GB11009 |
| PARP | CST | 9532 |
| Ki67 | CST | 12202 |
